# Efficacy of bortezomib in sarcomas with high levels of MAP17 (PDZK1IP1)

**DOI:** 10.18632/oncotarget.11475

**Published:** 2016-08-22

**Authors:** Marco Perez, Javier Peinado-Serrano, Jose Manuel Garcia-Heredia, Irene Felipe-Abrio, Cristina Tous, Irene Ferrer, Javier Martin-Broto, Carmen Saez, Amancio Carnero

**Affiliations:** ^1^ Instituto de Biomedicina de Sevilla, IBIS, Hospital Universitario Virgen del Rocio, Universidad de Sevilla, Consejo Superior de Investigaciones Cientificas, Seville, Spain; ^2^ Department of Vegetal Biochemistry and Molecular Biology, University of Seville, Seville, Spain; ^3^ Department of Medical Oncology, Virgen del Rocío University Hospital, Seville, Spain; ^4^ Department of Pathology, Virgen del Rocío University Hospital, Seville, Spain

**Keywords:** MAP17, bortezomib, PDX, sarcomas, biomarker

## Abstract

Sarcomas are malignant tumors accounting for a high percentage of cancer morbidity and mortality in children and young adults. Surgery and radiation therapy are the accepted treatments for most sarcomas; however, patients with metastatic disease are treated with systemic chemotherapy. Many tumors display marginal levels of chemoresponsiveness, and new treatment approaches are needed. MAP17 is a small non-glycosylated membrane protein overexpressed in carcinomas. The levels of MAP17 could be used as a prognostic marker to predict the response to bortezomib in hematological malignancies and in breast tumors. Therefore, we analyzed the expression of this oncogene in sarcomas and its relationship with clinico-pathological features, as well as tested whether it can be used as a new biomarker to predict the therapeutic response to bortezomib and new therapies for sarcomas. We found that the levels of MAP17 were related to clinical features and poor survival in a cohort of 69 patients with different sarcoma types, not being restricted to any special subtype of tumor. MAP17 expression is associated with poor overall survival (p<0.001) and worse disease-free survival (p=0.002). Cell lines with high levels of MAP17 show a better response to bortezomib *in vitro*. Furthermore, patient-derived xenografts (PDX) with high levels of MAP17 respond to bortezomib *in vivo*. Our results showed that this response is due to the lower levels of NFκB and autophagy activation. Therefore, we suggest that MAP17 is a new biomarker to predict the efficacy of bortezomib as a new therapy for sarcomas.

## INTRODUCTION

Sarcomas are rare tumors of mesenchymal origin [1, 2, 3] with high cancer mortality in children and young adults. The vast majority of sarcomas are sporadic, with the existence of a few genetically linked cancer syndromes. Furthermore, some types of environmental exposure have been associated with specific types of sarcoma [2, 3]. Taxonomical analysis of sarcomas has identified approximately 60 subtypes of sarcoma, as well as more than 50 benign tumor subtypes [4, 5]. Sarcomas are usually grouped into two broad categories according to molecular genetics: sarcomas harboring a diploid or nearly diploid karyotype and simple genetic driver alterations, such as Ewing's sarcoma, or sarcomas with a complex and imbalanced karyotype, such as osteosarcoma. The subgroups include very different clinical entities and are broadly drawn, not reflecting the genetic diversity among tumors of a given type or subtype or their diverse tumor biology [4].

Surgery is the standard approved treatment for most resectable sarcomas. Radiotherapy is selected for those unresectable or residual tumors after surgery. Chemotherapy is used in metastatic disease [6]. However, many tumors exhibit chemoresistance, and new treatments are needed. Systemic therapies for sarcomas include doxorubicin, gemcitabine, ifosfamide and the recently accepted drug trabectedin [7–10]. To date, few direct targets for therapy have been demonstrated in sarcomas, in contrast to those for epithelial cancers. The exceptions are GISTs, in which imatinib, the c-Kit kinase inhibitor, induces a partial response or stable disease in most sarcoma patients [4]. These findings support the hypothesis that widely diverse sarcoma tumors may share a dependence on a particular protein and that this marker may, therefore, be expected to be effective for all histological subtypes that are positive for this biomarker. We have recently extended this observation for CDK inhibition. We have found that sarcoma tumors with combined high levels of CDK4 mRNA and low levels of p16INK4a, respond to palbociclib (a CDK4 inhibitor) in a panel of PDX models *in vivo*, irrespective of the tumor type [11].

MAP17 (DD96, PDZKIP1), a membrane-associated protein with a molecular weight of 17 kD [12–14], is expressed only in the cells of the proximal tubule of the adult kidney [15–17]. It contains two transmembrane regions and a hydrophobic N-terminus encoding one PDZ-binding domain [18, 19]. Through this domain, MAP17 has been demonstrated to bind proteins containing PDZ domains, including PDZK1 (NHRF3), and other NHRF-related proteins, such as NaPiIIa and NHe3 [15, 17, 19]. MAP17 increases the cellular reactive oxygen species (ROS), which have been shown to enhance the malignant properties of tumor cells [20–22]. Tumor cells overexpressing MAP17 show increased proliferation, reduced apoptosis, increased formation in soft agar and increased growth of tumors in nude mice [20, 22]. However, the inhibition of ROS by antioxidant treatments can prevent the enhancement of the pro-tumorigenic properties of cells [14]. MAP17 is overexpressed in most human carcinomas and in other non-epithelial neoplasias such as glioblastomas or lymphomas [23]; additionally, its expression is associated with progression [17, 23, 24]. While adenomas and benign tumors, as well as normal tissues, rarely express MAP17, a high proportion (50 to 90%) of late-stage or metastatic tumors show high levels of MAP17, correlating with a more de-differentiated phenotype [17, 22, 24–26]. These findings highlight the relevance of this gene in the process of tumorigenesis and development of tumors.

MAP17, through an as-yet-unknown mechanism, increases ROS in cells. This ROS increase may be responsible, at least partly, for the enhanced malignant properties induced in tumor cells [33]. Because ROS is a potent proapoptotic insult, a further increase of ROS might switch the balance towards apoptosis. Thus, tumors with high MAP17 levels may respond to therapies increasing ROS. Therefore, MAP17 expression levels may be a suitable biomarker for prognosis. Cervix tumor patients treated with cisplatin and radiotherapy showed better survival if the tumor showed high levels of MAP17 [24, 27, 28]. Therefore, MAP17 is not only a marker for stage and malignant status but also may be a marker of response to therapies, inducing oxidative stress. In breast tumor cells, the MAP17 expression level determines the sensitivity to bortezomib by inhibiting the cytoprotective effects resulting from bortezomib-induced NFκB nuclear translocation and autophagy [29]. Furthermore, the inhibition of oxidative stress abolishes the MAP17-induced sensitivity to bortezomib. Therefore, high levels of MAP17 could be used for predicting bortezomib treatment responses in patients.

In this manuscript, we have explored the relevance of the presence of MAP17 in sarcoma tumors where the primary response is mainly achieved by treatments with radiotherapy. We found that, in contrast to laryngeal and cervix adenocarcinomas, the expression of MAP17 is a poor prognostic factor. Therefore, as we are looking for new therapeutic modalities for sarcomas, we have tested whether high levels of MAP17 may be a determinant in the response to bortezomib.

## RESULTS

### Clinical cohort description

The patient cohort with a clinical follow up had equal distribution by gender with 53% males and 47% females, with an average of 51 years at the time of diagnosis (Table 1). However, they showed a broad distribution in age, from 20 to 72 years. The histology was very variable with liposarcoma at 18.8% (n=13), undifferentiated pleomorphic sarcoma at 17.4% (n= 12) and leiomyosarcoma at 31.9% (n=22) as the most prevalent. Other types of minor represented sarcomas in our cohort were hemangiopericytoma at 4.3% (n=3), synovial sarcoma at 4.3% (n=3), neurogenic sarcoma at 4.3% (n=3), fibrosarcoma at 7.2% (n= 5), fibromyxoid sarcoma at 1.5% (n=1), angiosarcoma at 1.5% (n=1), and mesenchymal sarcomaat1.5% (n=1). These tumors were mostly metastatic at the time of diagnosis (76%), and the remainder was locally advanced. The treatment was doxorubicin 75 mg/m^2^ 3w × 6 cycles in all cases with half of the cases in combination with trabectedin 1.1 mg/m^2^ + doxorubicin 60 mg/m^2^ 3w × 6 cycles 49.3% (n=34). This cohort has been reported in Martin-Broto et al. [30].

**Table 1:** Clinical characteristics of the patients

Patients (N=69)	
**Gender**	
Male	53.6 (37)
Female	46.4 (32)
**Age** (years)	51 [20-72]
**Histology**	
Liposarcoma	18.8 (13)
Undifferentiated Pleomorphic Sarcoma	17.4 (12)
Haemangiopericytoma	4.3 (3)
Leiomyosarcoma	31.9 (22)
Synovial Sarcoma	4.3 (3)
Neurogenic Sarcoma	4.3 (3)
Fibrosarcoma	7.2 (5)
Unclassified Sarcoma	5.8 (4)
Fibromyxoid Sarcoma	1.5 (1)
Angiosarcoma	1.5 (1)
Condrosarcoma	1.5 (1)
Other	1.5 (1)
**Staging**	
Locally advanced	23.2 (16)
Metastatic	76.8 (53)
**Treatment**	
Doxorubicin 75 mg/m^2^ 3w × 6 cycles	50.7 (35)
Trabectedin 1.1 mg/m^2^ + Doxorubicin/3w 60 mg/m^2^ 3w × 6 cycles	49.3 (34)

Only for IHC analysis of MAP17 expression was this series completed with complementary samples (for which we do not have clinical information) used to assess MAP17 values in different sarcoma types (Supplementary Table S1).

### MAP17 Expression in human sarcoma

**Figure 1:**
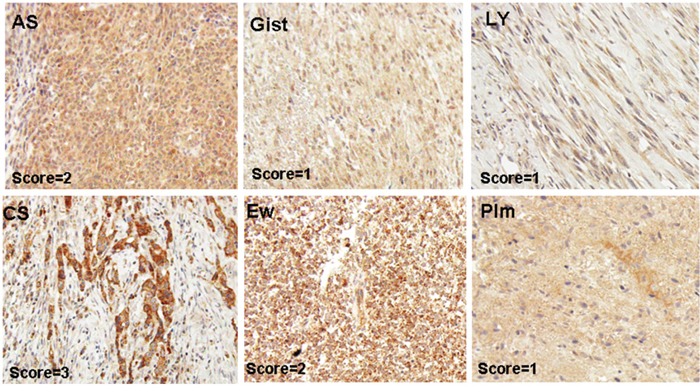
Representative pictures of MAP17-stained sarcoma samples Angiosarcoma (AS), Leiomyosarcoma (Ly), GIST (Gist), pleomorphic sarcoma (Plm), chondrosarcoma (CS), Ewing's sarcoma (EW).

**Figure 2:**
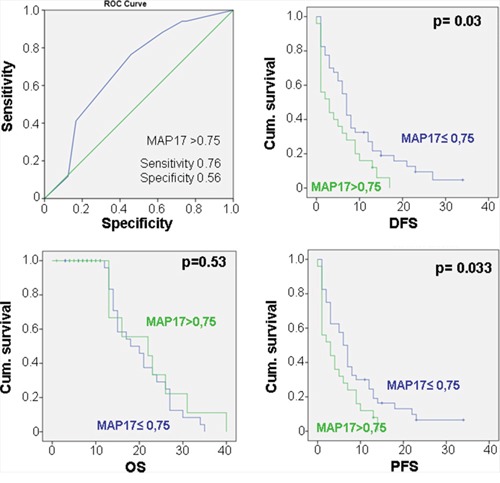
Relationship of the MAP17 levels with the clinical response in sarcomas ROC curve for MAP17 showing that, at the cut-off point of MAP17>0,75, the sensitivity=0.76 and thespecificity=0.56. Correlation of the MAP17expression was measured as a dichotomous variable: MAP17 high rates (>0,75) with overall survival (OS), disease-free survival (DFS) and progression free survival (PFS).

We analyzed 69 samples, for which we had clinical information, for MAP17 expression by immunohistochemistry (Figure 1), finding the percentage of tumors with high levels of expression as in other tumor types [17]. Using 0,75 as the cut-off level of MAP17 expression as depicted by the ROC curve (Figure 2), with a sensitivity = 0,76 and a specificity = 0,56, we found that high levels of MAP17 are predictive of a worse disease-free survival (DSF, p=0,03) and worse progression-free survival (PSF, p=0,033); however, there was no relevance regarding overall survival (OS) (Figure 2). These patients were treated with doxorubicin alone or with doxorubicin plus trabectedin. The analysis of the DFS, PFS or OS in these patients sorted by treatment scheme shows a similar trend in patients treated only with doxorubicin (Supplementary Figure S1A); in patients treated with the combination of doxorubicin plus trabectedin, MAP17 did not show any prognostic relevance (Supplementary Figure S1B).

**Figure 3:**
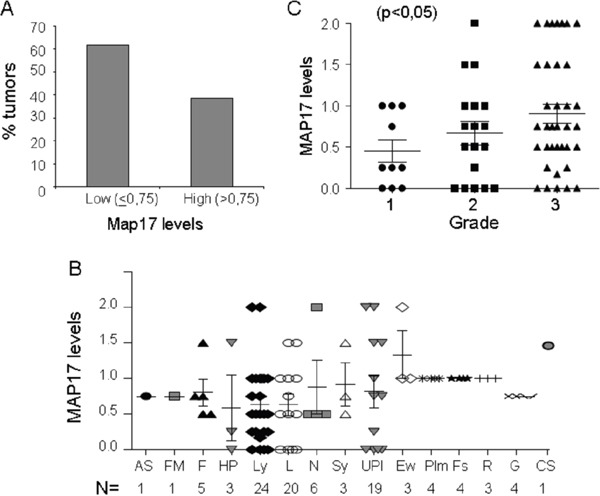
MAP17 expression in sarcoma tumors **A.** Overall distribution of MAP17-positive sarcomas. **B.** MAP17 level distribution among different sarcoma types. Angiosarcoma (AS), fibromyxoid sarcoma (FM), fibrosarcoma (F), hemangiopericytoma (HP), leiomyosarcoma (Ly), liposarcoma (L), neurogenic sarcoma, MPNST (N), synovial sarcoma (Sy), undifferentiated pleomorphic sarcoma (UPl), Ewing's sarcoma (Ew), rhabdomyosarcoma (R), fusocellular sarcoma (Fs), GIST (G), pleomorphic sarcoma (Plm), and chondrosarcoma (CS). **C.** Map17 levels correlate with tumor grade in sarcoma. The mean values are represented for grade 1 (=0,45), grade 2 (=0,66) and grade 3 (0,90). ANOVA was performed to establish the statistical association between the MAP17 protein levels and grade of the tumor (p<0,05).

One hundred one tumor samples were analyzed for MAP17 expression by immunohistochemistry. Of the 101 samples, 39% showed higher levels of MAP17 protein expression and were considered positive for MAP17 expression (Figure 3A) according to the previous ROC curve performed. The distribution of tumors with MAP17 was broad and was not restricted to any specific sarcoma type (Figure 3B), but there was a direct correlation of the levels of MAP17 with tumor grade (ANOVA, p<0.05) (Figure 3C), which was statistically significant. The surrounding normal tissue did not express MAP17 or expressed very low levels.

The analysis of the impact on DFS, PFS or OS on specific sarcoma types such as liposarcoma, undifferentiated or leiomyosarcoma, showed a similar trend to the total population comprising all sarcoma types (Supplementary Figure S2). However, the small number of cases precludes any strong conclusion. Furthermore, the analysis of databases for the expression of MAP17 in other types not sufficiently represented in our cohort, such as osteosarcoma or Ewing's sarcoma with a high MAP17 level, also shows a worse prognosis in either OS or metastasis-free survival (Supplementary Figure S3).

Therefore, our data showed that 40% of sarcomas display high levels of MAP17 protein at the time of diagnosis, unrelated to the specific tumor type. High levels of MAP17 are related to a higher grade and are predictive of a poor prognosis in patients with sarcoma.

Furthermore, multivariate analysis indicated that MAP17 is an independent predictor of the sarcoma outcome (Table 2).

**Table 2:** Multivariate analysis. MAP17 is an independent predictor of other clinical variables

	DFS		PFS		OS	
	Pearson correlation	p-value	Pearson correlation	p-value	Pearson correlation	p-value
**MAP17**	−0.268	0.015	−0.268	0.016	−0.142	0.129
**Radiotherapy**	−0.155	0.134	−0.143	0.154	0.162	0.123
**Differentiation**	−0.216	0.06	−0.167	0.116	−0.245	0.038
**Metastatic disease**	−0.191	0.085	−0.125	0.187	−0.207	0.069
**T4 extension**	−0.114	0.208	−0.157	0.130	−0.207	0.069

### The response to bortezomib in sarcoma cell lines correlates with MAP17 Levels

Because these patients with high MAP17 showed a worse prognosis, we wondered whether we can find an alternative treatment for these patients. To this end, we planned to test bortezomib, which showed efficacy in breast tumor cells if MAP17 is overexpressed [29]. Thus, we first analyzed the role of MAP17 levels in sarcoma cell lines.

**Figure 4:**
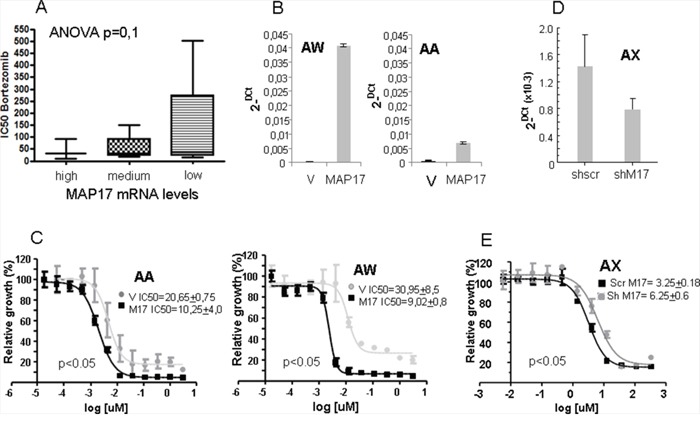
Correlation of the MAP17 levels with the sensitivity to bortezomib **A.** Correlation of the MAP17 levels with the sensitivity to bortezomib in a panel of 16 sarcoma cell lines. The cell lines were subdivided according to the levels of MAP17: no or barely detectable levels of MAP17 mRNA= **Low** (BP, CE, CP0024, SAOS2, SK-UT-1, HT-1080 and CP0038); cells with low but clearly detectable levels of MAP17 mRNA= **medium** (AA, AW, BG and BD); and high levels of expression= **high** (BC, AZ, AX, SW872 and 93T449). The analysis of the correlation of MAP17 mRNA levels and IC50 to bortezomib was performed with ANOVA, p=0,1. **B.** Levels of MAP17 mRNA in cell lines ectopically expressing MAP17 cDNA (MAP17) or vector only (V). The data show the average levels of MAP17 mRNA by Q-RT-PCR, performed in three independent determinations. **C.** Curves showing the IC50 of cell lines ectopically expressing MAP17 cDNA (MAP17) or vector only (V). IC50 in the inset is the average ± SD from 4 independent experiments performed in triplicate. **D.** Decrease of the levels of MAP17 mRNA by specific expression of shmRNS against MAP17 in the sarcoma cell line AX. The data show the average levels of MAP17 mRNA by Q-RT-PCR, performed in three independent determinations. **E.** Curves showing the IC50 of cell lines ectopically expressing MAP17 sh mRNA (shM17) or scramble shRNA as control (Scr M17). IC50 in the inset is the average ± SD from 3 independent experiments performed in triplicate.

To explore the effect of MAP17 on the response to bortezomib, we used a panel of 16 sarcoma cell lines of heterogeneous origin [11, 31, 32]. We treated them at different concentrations of bortezomib and obtained an IC50 value for each one. All of the responses were in the nM range (Table 3), distributed however in a 50-fold range, between 10 and 500 nM. In parallel, we measured the basal levels of MAP17 mRNA in all cell lines (Table 3) and found different expression levels between them. We distributed the cell lines into three groups according to the MAP17 mRNA level: those with barely detectable MAP17 mRNA (BP, CE, CD0024, SAOS2, SK-UT-1, HT-1080 and CP0038), those with low but clearly detectable levels of MAP17 mRNA (AA, AW, BG and BD) and those with high levels of expression (BC, AZ, AX, SW872 and 93T449). The analysis of the correlation of the MAP17 mRNA levels and IC50 to bortezomib showed a trend (ANOVA, p=0,1) indicating that cells with higher levels of MAP17 are more sensitive to bortezomib than cells without MAP17 (Figure 4A).

**Table 3:** IC50 to bortezomib of the panel of sarcoma cell lines used in this study and its correlation to the levels of MAP17 mRNA measured by RT-QPCR

Cell line	Tumor of origin	IC50 Bortezomib (μM)	MAP17 (2-ΔCT)
93T449	Liposarcoma	35,9±3,75	0,000182199
AA	Leiomyosarcoma	20,65±0,75	6,21882E-05
AW	Liposarcoma	30,95±8,5	0,00011437
AX	Liposarcoma	19,3±5,2	0,000534323
AZ	Fibrosarcoma	92,89±0,92	0,001050201
BC	MPNST	32,5±9,8	0,00025699
BD	Ewing's Sarcoma	149,7±17,42	0,000111012
BG	Myxoid Fibrosarcoma	28,99±5,8	7,64036E-05
BP	Osteosarcoma	502±56,6	2,32244E-05
CE	Rhabdomyosarcoma	15,53±2,9	4,55244E-05
CP0024	Leiomyosarcoma	30,75±5,46	2,84148E-05
CP0038	Leiomyosarcoma	43,65±9,78	2,52213E-05
HT1080	Fibrosarcoma	15,88±2,37	1,68955E-05
Saos-2	Osteosarcoma	52,78±9,5	1,30192E-05
SKUT1	Uterine Leiomyosarcoma	10,95±2,46	4,58532E-05
SW872	Liposarcoma	10,13±2,36	0,000262208

IC50 is the average of 3 independent experiments performed in triplicate. To measure human MAP17 RNA expression, real-time PCR was performed using a ABI 7900HT (Applied Biosystems), and we used GADPH as internal control/reference. Quantitative and statistical analyses of the QPCR data were calculated using Applied Biosystem RQ Manager 1.2.1 software. We presented the 2eΔΔCT data. The levels of MAP17 mRNA presented are the average of the mRNA levels obtained in three independent determinations performed in triplicate samples.

To functionally confirm these data, we overexpressed MAP17 cDNA in 2 sarcoma cell lines with very low expression, AA and AW (Figure 4B), and calculated a new IC50 to bortezomib in these cell lines and compared to parental cells expressing empty vector only. We found that the ectopic expression of MAP17 increases the sensitivity to bortezomib 2-3 fold (Figure 4C). To fully confirm these results, we reduced the levels of MAP17 in the AX sarcoma cancer cell line, which shows high endogenous levels of MAP17. We overexpressed an shRNA against MAP17 and reduced the levels of MAP17 to 50% (Figure 4D), and then we tested the bortezomib sensitivity (Figure 4E). We observed a 50% reduction in the sensitivity, comparable to the 2-fold increase observed in the cell lines with enforced overexpression of MAP17.

### Sarcomas overexpressing MAP17 are more sensitive to bortezomib *in vivo*

**Figure 5:**
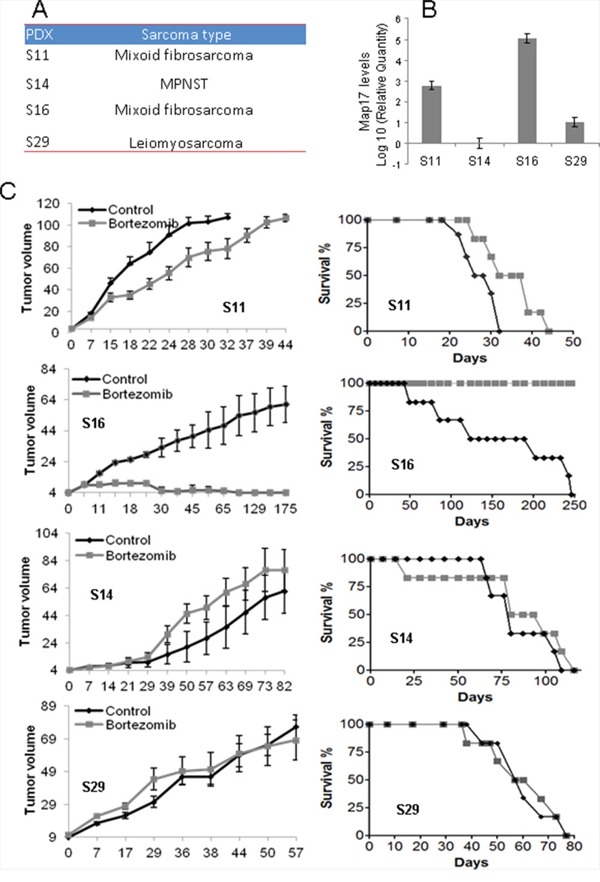
Effect of bortezomib on sarcoma tumors *in vivo* **A.** PDX models used in this study. **B.** Levels of MAP17 mRNA in each model. **C.** Response to bortezomib. The left graph shows the average ±SD of tumor growth. The right graph shows the survival of the mouse cohorts with sacrifice by humane endpoint when the tumor reaches 1000 mm^3^. The sarcomas were subcutaneously engrafted and grown until all of the tumors reached 20 mm^3^ in volume. The mice were then treated for 4 weeks (5 days/eek). The cohorts of mice were either treated with bortezomib (1.0 mg/kg body weight, in 0.9% NaCl) or saline serum (0.9% NaCl). The mice were monitored daily for signs of distress and were weighed twice a week. The tumor size was measured using a caliper according to the following equation: tumor volume = [length × width^2^]/2. The experiments were terminated when the tumor reached 1000 mm^3^.

Therefore, we decided to test the response of sarcomas to bortezomibin patient-derived xenografts, PDXs, *in vivo*. Of a panel of sarcoma PDXs [11], we selected 4 models (Figure 5A) with different levels of MAP17 (Figure 5B). Twelve mice were implanted for each PDX model and were randomly distributed for the treatment with bortezomib or solvent alone (PBS). We found that the PDX with high levels of MAP17 showed a better response to bortezomib (Figure 5C). The tumor disappeared from the flank of all mice treated with bortezomib, and the mice survived. The PDX model S11, with still significant but low levels of MAP17, partially responded to bortezomib by decreasing the tumor growth rate and increasing survival by 25% approximately (Figure 5C). However, the models with low or no MAP17 (S14 and S29) did not respond to bortezomib (Figure 5C). Thus, our data *in vivo*, in PDX models of sarcoma, support our data *in vitro* and suggest that bortezomib may be a suitable therapy for sarcoma patients with high MAP17 at diagnosis.

**Figure 6:**
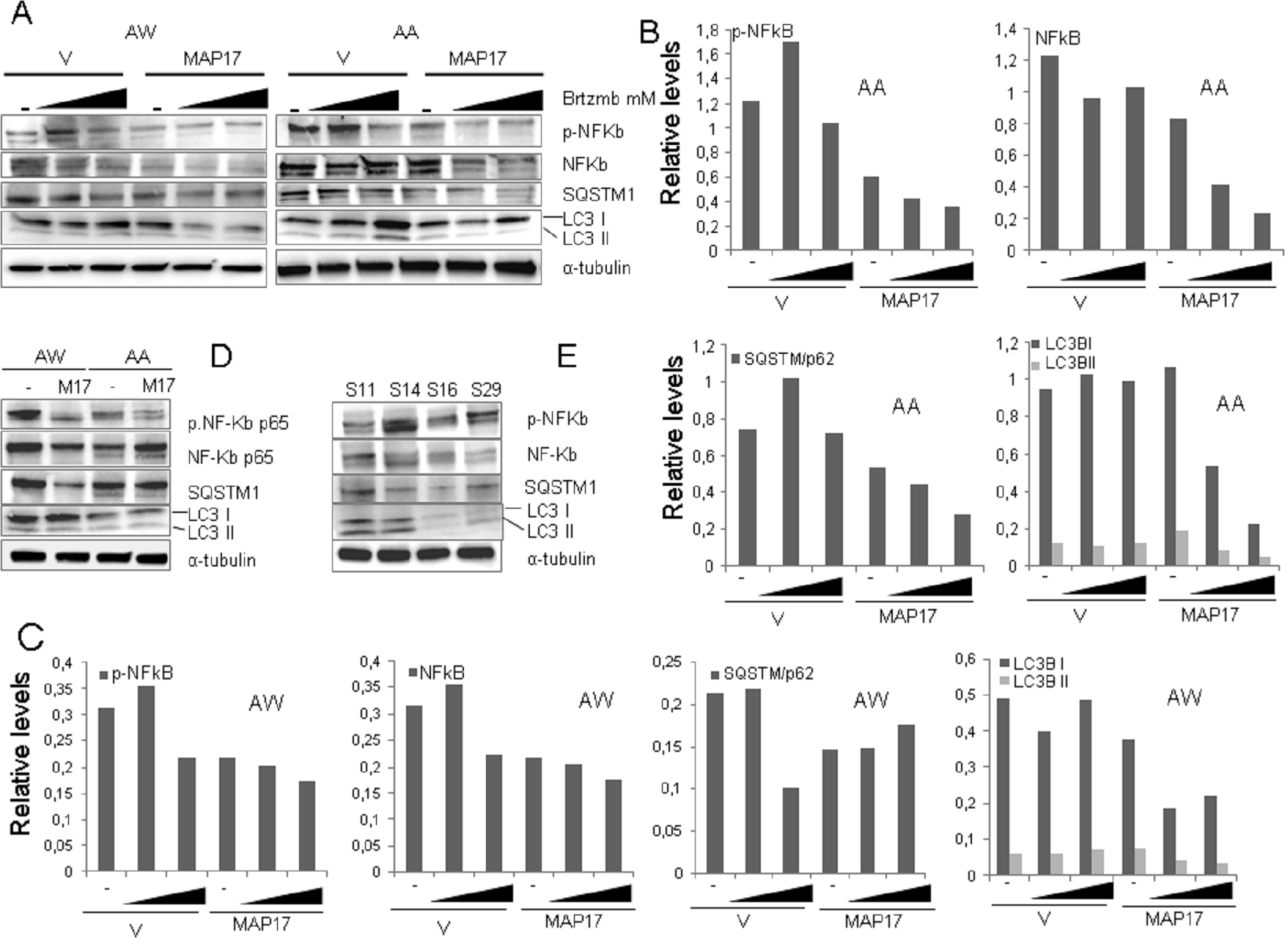
Levels of NFκB and autophagy in sarcoma tumors or cell lines expressing MAP17 **A.** Western blots showing the levels of different basal protein levels in sarcoma PDX models. NFκB-p65 phosphorylated at Ser536, total NFκB-p65, LC3B and p62. Treatment with bortezomib was performed for 24 hrs. **B.** Quantification of Figure 6A, cell line AA. **C.** Quantification of Figure 6A, cell line AW. **D.** Western blots showing the levels of different proteins in AA and AW sarcoma cell lines overexpressing MAP17 or empty vector only. NFκB-p65 phosphorylated at Ser536, total NFκB-p65, LC3B and p62. Quantification is shown in Figure 4A. **E.** Western blots showing the levels of different proteins in AA and AW sarcoma cell lines overexpressing MAP17 or empty vector only, in response to bortezomib (+) or to solvent only (−). NFκB-p65 phosphorylated at Ser536, LC3B and p62 levels are shown. Quantification is shown in Figure 4B.

We have previously shown that MAP17 prevents cytoprotective NFκB activation and autophagy induced by bortezomib in breast tumor cells [29]. Therefore, we have tested whether these molecular markers correlate also in our PDX models prior to treatment (Figure 6A). S16, the PDX model that responds to bortezomib, showed lower levels of endogenous active NFκB (measured as p65 phosphorylated at Ser536) and autophagy (measured as p62 and/or LC3 increase), thus confirming the mechanistic role of these factors in the bortezomib response. Similar data could be observed in the AA and AW cell lines, where the overexpression of MAP17 reduces NFκB activation and autophagy (Figure 6B, Supplementary Figure S4A). Moreover, we observed that MAP17 prevents the cytoprotective activation of NFκB and autophagy induced by bortezomib (Figure 6C, Supplementary Figure S4B) in sarcoma cell lines, a finding that has been reported previously for breast tumor cells [29]. Therefore, the inhibition of these protective pathways induced by MAP17 may explain the increase in sensitivity to bortezomib found *in vivo*.

## DISCUSSION

High MAP17 levels correlate with a higher grade and poorer differentiation of tumors; therefore, therapies that can counteract MAP17 expression may lead to the promising application of known treatments to tumors with a poor prognosis. Sarcoma tumors that express MAP17 have a poor prognosis independent of the tumor origin. However, sarcoma cell lines and PDX tumors *in vivo* with high levels of MAP17 respond to the proteasome inhibitor bortezomib (Velcade, PS-341), approved by the FDA for the treatment of mantle cell lymphoma and multiple myeloma [33, 34]. Thus, our work provides first evidence that certain patients may benefit from this therapy that could be newly applied to second- or third-line sarcoma patients, currently without any other therapeutic option.

MAP17 maintains a functional relationship with the proteasomal degradation pathway [29]. Used as a “seed”, MAP17 was found to be linked to proteins related to degradation pathways, mainly ubiquitination- and sumoylation-related pathways. We found that multiple myeloma patients with higher MAP17 mRNA levels respond better to bortezomib and exhibit prolonged survival [29]. We also showed that MAP17 determines the bortezomib sensitivity by inhibiting the cytoprotective effects related to bortezomib-induced NFκB nuclear translocation and autophagy. Therefore, high levels of MAP17 could be used to select some patients for which bortezomib is not currently indicated, such as sarcoma, but who could benefit from this therapy.

We found that the MAP17-dependent increase in sensitivity in sarcoma was correlated with lower levels of phosphorylated NFκB and autophagy measured as p62/LC3II, further supporting the relevance of these two pathways in MAP17 increased sensitivity. Therefore, the inhibition of autophagy or NFκB pathways may also cooperate with bortezomib in the response to sarcomas. It will be of interest to test the response to bortezomib, or other proteasome inhibitors [35], in combination with autophagy or NFκB inhibitors in MAP17-expressing sarcomas.

MAP17 is mainly expressed in advanced tumor stages but not in normal tissue, benign tumors (adenomas) or early-stage neoplasias [23]. In all human tumors, MAP17 is an independent prognosis marker. MAP17 overexpression correlates with advanced stages in ovarian, cervical, laryngeal and prostate tumors [23]. Mammary tumor cells that express MAP17 show an enhanced tumor phenotype, which is characterized by enhanced proliferative capabilities [22]. The increased tumorigenic properties caused by the expression of MAP17 are associated with high levels of ROS, and the inhibition of antioxidants decreases their malignant properties. Inhibition of ROS with antioxidant treatments also inhibits the effect of MAP17 on NFκB activation and autophagy, restoring resistance to bortezomib and indicating that ROS are involved in this process. It has been shown that bortezomib favors the unfolded protein response (UPR), which is activated in response to alterations in the ER physiological environment [36–38]. This ER stress stimulates ROS production, which alters the responses to bortezomib treatment in patients with MCL [38] and MM [39]. The MAP17-induced increase in ROS is also functionally related to NHeRFs. Inhibition of these transporters by furosemide impairs the response to bortezomib, thus leading to the recovery of bortezomib resistance in MAP17-overexpressing cells [29]. Currently, there are several clinical trials with bortezomib as monotherapy or in combination with other drugs in sarcoma patients [40, 41]. It will be interesting to analyze the results of the trials in relation to MAP17 expression.

In sarcoma, MAP17 is a marker for poor DFS irrespective of the tissue type, indicating that MAP17 has no role in the response to current treatments, including doxorubicin alone or in combination with trabectedin. However, in laryngeal and cervical tumors, MAP17 is a potent marker for better prognosis, including longer overall survival [24, 27, 42]. In cervical cells, the overexpression of MAP17 makes these cells more sensitive to cisplatin and/or radiotherapy [24, 27], explaining the enhanced sensitivity. However, sarcomas are primarily managed by surgery and/or radiation, being this last treatment modality mainly used in the neo-adjuvant or adjuvant setting in combination or not with systemic therapies [6]. We do not have adequate information to study the role of MAP17 as a prognostic marker in un-resectable tumors treated with radiotherapy, alone or in combination with other chemotherapies such as ifosfamides, but it certainly deserves attention. Our own preliminary data suggest that sarcoma cells expressing MAP17 are also more sensitive to ROS-inducing therapies such as cisplatin (unpublished observation).

High MAP17 levels correlate with a higher grade and poorer differentiation of sarcoma, and tumors that express MAP17 at diagnosis have a poor prognosis independent of the tumor origin. However, sarcoma cell lines and PDX tumors *in vivo* with high levels of MAP17 respond to the proteasome inhibitor bortezomib [33, 34]. Thus, our work provides the first evidence that certain patients may benefit from this therapy that could be newly applied to second- or third-line sarcoma patients, currently without any other therapeutic option.

## MATERIALS AND METHODS

### Tumor samples for immunohistochemistry studies

The cohort of 69 patients for immunohistochemistry studies and the correlation of clinico-pathological features (Table 1) were obtained from the Sarcoma Research Spanish Group Trial 20, Geis 20 [30]. For the study of the MAP17 expression of different sarcoma types (Figure 3B), this cohort was complemented with 24 extra samples (Supplementary Table S1) obtained from tumor tissues obtained from the surgical resection of sarcomas performed at University Hospital Virgen del Rocio (Seville, Spain). All of the patients provided written informed consent according to a protocol approved by the local ethics committee (CEI 2013/PI002). All tissue samples and patient information were treated according to the Declaration of Helsinki.

### Immunohistochemistry

Three-micrometer slices were sectioned from the TMA block and were applied to coated, immunochemistry slides (DAKO, Glostrup, Denmark). The slides were baked overnight in a 56°C oven, deparaffinized in xylene for 20 min, rehydrated through a graded ethanol series and washed with PBS. A heat-induced epitope retrieval step was performed by heating a slide in a solution of sodium citrate buffer at pH 6.5 for 2 min in a conventional pressure cooker. After heating, the slides were incubated with proteinase K for 10 min and were rinsed in cool running water for 5 min. Endogenous peroxide activity was quenched with 1.5% hydrogen peroxide (DAKO) in methanol for 10 minutes, and incubation with the primary antibodies anti-MAP17 (1:4) [14, 20, 22, 25] was performed for 40 min. After incubation, immunodetection was performed with the EnVision (DAKO, Glostrup, Denmark) visualization system using diaminobenzidinechromogen as the substrate, according to the manufacturer's instructions. Immunostaining was performed in a TechMate 500 automatic immunostaining device (DAKO) and was measured through a double-blind visual assessment using microscopic observation according to the anatomopathological experience of pathologists. Sample scoring was performed by semiquantitative microscopic analysis, considering the signal intensity. We used the score obtained by the intensity levels (1, 2 or 3). The threshold used is the score of 0,75, obtained by ROC curve as the most relevant to establish as a dichotomous variable.

### Tumor samples for PDX generation

Tumor tissues were obtained from the surgical resection of sarcomas performed at University Hospital Virgen del Rocio (Seville, Spain). All of the patients provided written informed consent according to a protocol approved by the local ethics committee (CEI 2013/PI002). The experiments were performed according to the European guidelines for laboratory animal care. This study was approved by the IBIS Institutional Animal Care and Use Committee.

### PDX generation

Sarcoma tissue samples were obtained from a single tumor area and were preserved in Dulbecco's modified Eagle's medium nutrient mixture/F10 (DMEM/F10; Sigma) containing 10% fetal bovine serum, penicillin, streptomycin and amphotericin B (100 mg/ml each; Sigma). The samples were maintained for less than 2 hours in cell culture medium at room temperature before implantation. Each tissue was divided into 2 parts. One part was frozen, and the remaining part was cut into small fragments of 2-3 mm in diameter to be used for subcutaneous implantation into 6-week-old Foxn1nu athymic nude female mice (Harlan Laboratories, Netherlands). Upon reaching a size of 1,500 mm^3^, the mice were euthanized, and the tumors were re-grown in a similar fashion to perform the indicated experiments.

### *In vivo* treatments

To initiate the experiments, each sample was xenografted into mice. Once the tumors reached 1500 mm^3^, they were harvested, cut into 2×2×2-mm blocks and implanted. The experiments were performed using cohorts of 6 animals. the mice were randomly allocated to the drug-treated and control-treated (solvent only) groups; once the tumor grew to 20 mm^3^, the mice were treated for 4 weeks (5 days/week). The cohorts of mice were treated with either bortezomib (1.0 mg/kg body weight, in 0.9% NaCl) or saline serum (0.9% NaCl). The mice were monitored daily for the signs of distress and were weighed twice a week. The tumor size was measured using a caliper according to the following equation: tumor volume = [length × width^2^]/2. The experiments were terminated when the tumor reached 1000 mm^3^.

### Western blot analyses

Western blot analyses were performed as previously described [43, 44]. Briefly, the cells were washed twice with PBS and were lysed via sonication in lysis buffer (50 mM Tris-HCl, pH 7.5; 1% NP-40; 1 mM Na_3_VO_4_; 150 mM NaCl; 20 mM Na_4_P_2_O_7_; 100 mM NaF; 1% Na-deoxycholate; 0.1% SDS; 1 mM EDTA; phosphatase inhibitor cocktail (Sigma) and protease inhibitor cocktail (Sigma)). The samples were separated on 6–15% SDS-PAGE gels, transferred to nitrocellulose membranes (Protran BA83; Whatman) and immunostained. The following primary antibodies and dilutions were used: anti-NFκB-p65 (1:2000; Abcam #ab16502), anti-NFκB-p65 (phospho Ser536) [93H1] (1:1000; Cell Signaling #3033), anti-LC3B (1 μg/ml; Abcam #ab48394), anti-SQSTM1/p62 (1:20000; Abcam #Ab 109012) and anti-α-tubulin (1:5000; Sigma 9026). Horseradish peroxidase-labeled rabbit anti-mouse (Amersham, diluted 1:3000) and goat anti-rabbit (Abcam, #6721, diluted 1:3000) secondary antibodies were used. The proteins were visualized using an ECL detection system (Amersham Biosciences).

### Quantitative mRNA determination

Total RNA was isolated via cell lysis in Qiazol reagent using an RNA Mini Kit (Qiagen, Inc.). First-strand cDNA synthesis was performed using 2.0 μg of RNA, random primers, a dNTP mix and Multiscribe Reverse Transcriptase in a total volume of 50 μl (High Capacity Transcription Kit, Applied Biosystems). The conditions used for RT-PCR were as follows: 10 min at 25°C, 120 min at 37°C, and 5 min at 95°C. To measure human MAP17 expression, real-time PCR was performed using an ABI 7900HT PCR system (Applied Biosystems). The qPCR reactions were performed in 384-well plates via TaqMan Gene Expression Assays (Applied Biosystems). Gadph expression was examined as an internal control. The relative mRNA quantities were expressed as 2-ΔCt. The relative mRNA quantification and statistical analysis of qPCR data were conducted using RQ Manager 1.2.1 software (Applied Biosystems).

### Human primary sarcoma cell lines and culture conditions

The sarcoma cell lines used in this study were previously characterized [11, 31, 32]. The cells were maintained as a subconfluent monolayer in F-10 medium (Sigma) supplemented with 10% FBS, penicillin-streptomycin antibiotics (Sigma) and Fungizone (Amphotericin B, Sigma). Each cell line was cultured at 37°C and 95% humidity in 5% CO_2_ under conditions of O_2_ levels, culture medium and supplements indicated in the provider's instructions.

### Transfection

Subconfluent AA and AW cells were transfected using the Lipofectamine method (Effectene, Qiagen) with 0.4 μg of the empty mammalian expression plasmid pBabepuro or pBabepuro containing either the wild-type MAP17 gene [45]. At 24 hours after transfection, the cell lines were cultured in F-10 medium supplemented with 10% FBS and 2 μg/ml puromycin.

### Cytotoxicity assay

Bortezomib was freshly prepared as a 30-mM stock solution in sterilized deionized water for each experiment. Bortezomib was applied to a 96-well master plate at decreasing concentrations of 1/3, such that 300 μM was the highest concentration studied. The cell lines were seeded in 96-well plates (5,000-10,000 cells per well, depending on the cell size). At 24 hours after seeding, treatment was applied for 96 hours. Cell proliferation was determined by the MTT assay and was confirmed by crystal violet staining [46]. The IC50 was calculated using GraphPad Prism software.

### Statistical Analysis and Definitions

Kaplan-Meier method was used for survival analysis, using the Cox proportional hazards model to adjust for the explanatory variables, obtain the p-values and estimate the HR. Multivariate logistic regression was used to obtain the odds ratios (ORs) and confidence intervals (CI 95%). Pearson's correlation measured the dependence between the quantitative variables. Receiver operating characteristic (ROC) curve analysis was performed to assess the MAP17 cutoff point, which we checked using the optimal Youden index-based point. In addition, the log-rank test was used to compare the survival distributions between the high and low MAP17 levels. Statistical calculations were performed using SPSS 22.0 software. OS was defined as the length of time from the date of diagnosis until the date of the last medical record.

## SUPPLEMENTARY MATERIAL FIGURES AND TABLE


