# Supplementary material for: Efficacy of bortezomib in sarcomas with high levels of MAP17 (PDZK1IP1)
Source: Oncotarget. 2016 Aug 22;7(41):67033–46. doi: 10.18632/oncotarget.11475 (PMC5341855; doi:10.18632/oncotarget.11475)
Supplement: Supplementary file 1 [file oncotarget-07-67033-s001.pdf]

Efficacy of bortezomib in sarcomas with high levels of MAP17 (PDZK1IP1)

SUPPLEMENTARY FIGURES AND TABLE

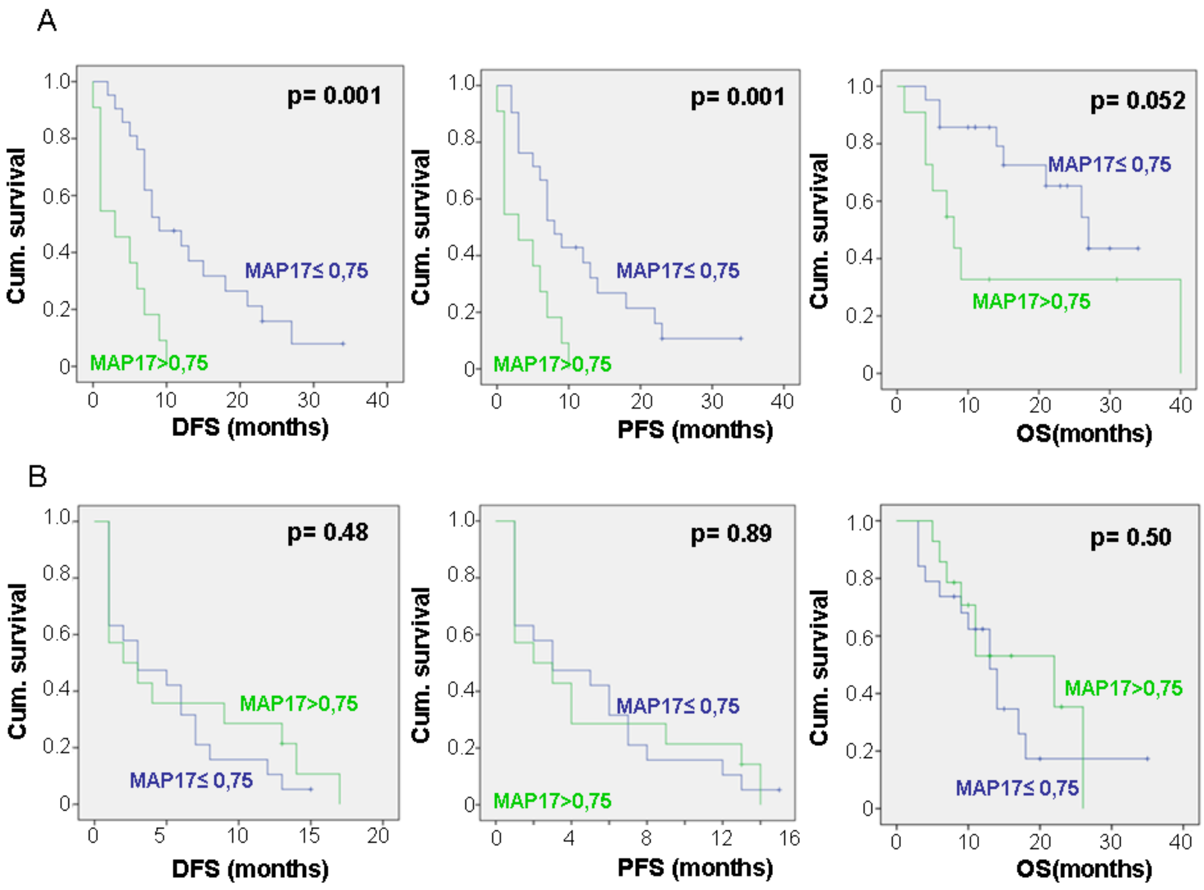

Supplementary Figure S1: Relationship of the MAP17 levels with the clinical response in sarcomas according to the treatment. A. Correlation of the MAP17 expression measured as a dichotomous variable: MAP17 high rates (>0,75) with overall survival (OS), disease-free survival (DFS) and progression-free survival (PFS) in patients treated only with doxorubicin. N= 35. **B.** Correlation of the MAP17 expression measured as a dichotomous variable, with overall survival (OS), disease-free survival (DFS) and progression-free survival (PFS) in patients treated with a combination of doxorubicin plus trabectedin. N= 34.

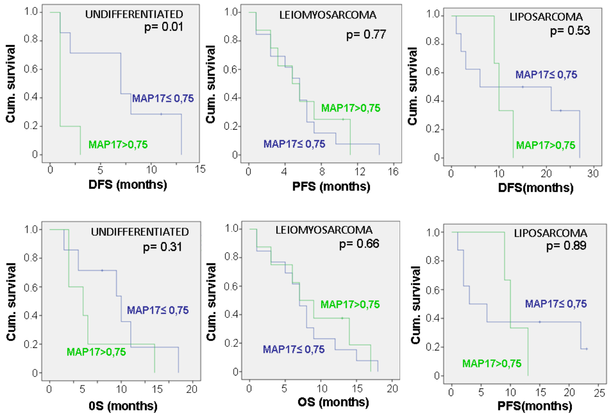

Supplementary Figure S2: Relationship of the MAP17 levels with the clinical response in specific types of sarcomas. Correlation of the MAP17 expression measured as a dichotomous variable: MAP17 high rates (>0,75) with overall survival (OS), disease-free survival (DFS) and progression-free survival (PFS). Analysis was performed in the only three types of sarcomas most represented n>10 in our patient cohort.

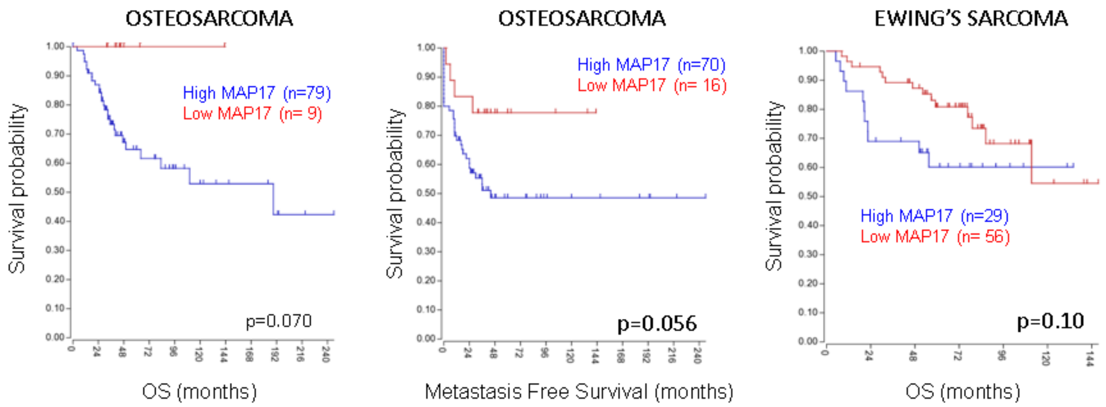

Supplementary Figure S3: Relationship of the MAP17 levels with the clinical response in osteosarcomas and Ewing's sarcomas. Correlation of the MAP17 expression measured as a dichotomous variable in cohorts reported by transcriptomic analysis. Ewing's Database was obtained from GSE17679, from Savola et al [47]; the osteosarcoma database was obtained from Kuijjer et al [48]. Analysis was performed by *R2: Genomics Analysis and Visualization Platform*, and the MAP17 cut-off point was extracted by scan with overall survival (OS), disease-free survival (DFS) and metastasis-free survival (MFS).

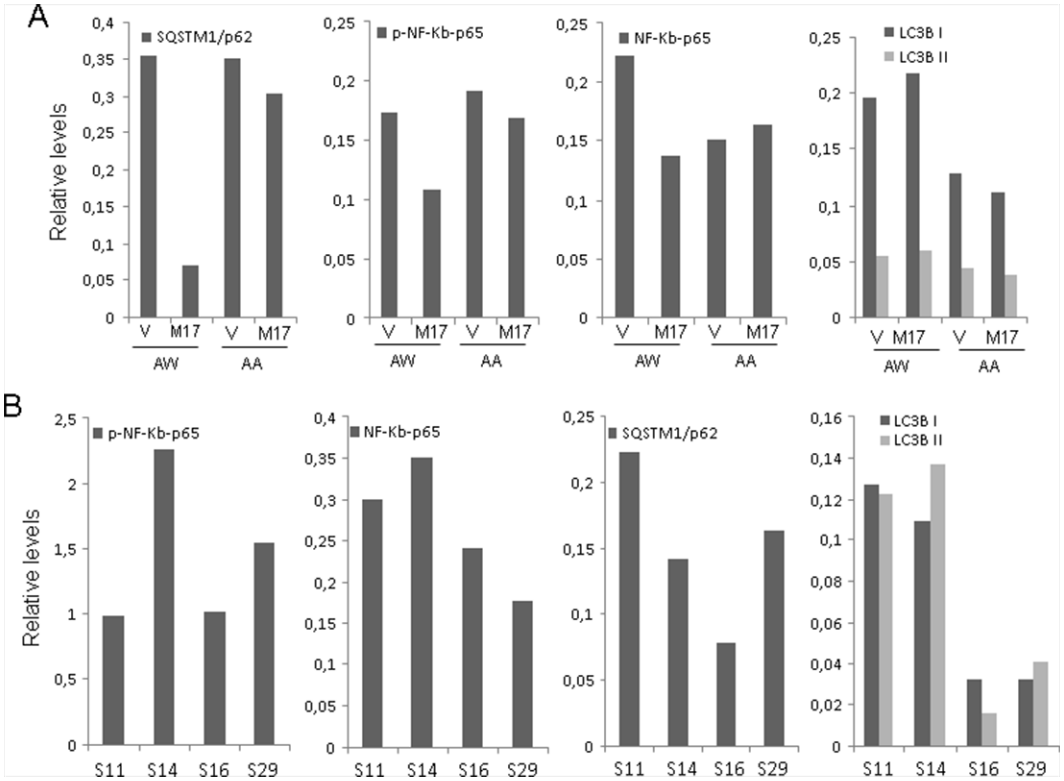

Supplementary Figure S4: A. Quantification of the levels of NFκB-p65 phosphorylated at Ser536, LC3B and p62 levels related to the total a-tubulin protein. Quantification corresponding to Figure 6D; **B.** Quantification of the levels of NFκB-p65 phosphorylated at the Ser536, LC3B and p62 levels related to the total a-tubulin protein. Quantification corresponding to Figure 6E.

Supplementary Table S1: Complementary samples used to assess MAP17 values in different sarcoma types

Angiosarcoma (AS) n= 1
Leiomyosarcoma (Ly) n= 3
Liposarcoma (L) n= 2
Neurogenic sarcoma (N) n= 3
Rhabdomyosarcoma (R) n= 3
Fusocellular sarcoma (Fs) n= 4
GIST (G) n= 3
Pleomorphic sarcoma (Plm) n= 4
